# Colonoscopy After Acute Diverticulitis: Low Yield in Uncomplicated Disease, Higher Risk in Complicated Cases

**DOI:** 10.7759/cureus.107101

**Published:** 2026-04-15

**Authors:** Ahmed Gerwash, Jia Hui Loo, Najiyah Khanom, Jane Lai, Stephen O Agboro, Ahmed Hammad

**Affiliations:** 1 General Surgery, University Hospital Lewisham, London, GBR

**Keywords:** colorectal cancer, complicated diverticulitis, diagnostic yield, sigmoid diverticulitis, uncomplicated diverticulitis, colonoscopy

## Abstract

Background: The optimal role of routine colonoscopy following an episode of acute diverticulitis remains a subject of ongoing debate, with contemporary guidelines increasingly advocating for a selective, risk-based approach. This study aimed to evaluate the diagnostic yield of colonoscopy after diverticulitis and to assess the influence of disease complexity on the detection of colorectal neoplasia.

Methods: A retrospective cohort study was conducted on 268 patients with CT-confirmed acute diverticulitis. Colonoscopy was performed in 168 patients (62.7%). Patients were categorized into uncomplicated (n=179, 66.8%) or complicated (n=89, 33.2%) diverticulitis based on established radiological and clinical criteria. Colonoscopic findings were classified as normal mucosa, diverticulosis, benign polyps, strictures, or colorectal cancer. The association between disease complexity and colonoscopic findings was assessed using chi-square testing.

Results: Among the 168 patients who underwent colonoscopy, the predominant finding was diverticulosis (n=137, 81.5%), followed by normal mucosa (n=16, 9.5%), benign polyps (n=10, 6.0%), strictures (n=4, 2.4%), and colorectal cancer (n=1, 0.6%). Of these, 115 patients (68.5%) had uncomplicated diverticulitis and 53 (31.5%) had complicated diverticulitis. All malignant pathology (n=1, 0.6%) occurred exclusively in the complicated group, yielding a cancer detection rate of 1.9% (1/53) in complicated diverticulitis versus 0% (0/115) in uncomplicated diverticulitis. Benign polyps were similarly distributed between groups, while strictures were more frequent in complicated disease. Normal colonoscopy findings were more common after uncomplicated episodes.

Conclusion: Colonoscopy performed after uncomplicated diverticulitis demonstrated a very low diagnostic yield for colorectal cancer. Conversely, malignancy was exclusively identified in patients with complicated disease. These findings support a selective strategy, prioritizing colonoscopy for patients with complicated diverticulitis or those with additional risk factors, rather than a universal post-diverticulitis endoscopic evaluation.

## Introduction

Diverticular disease is a common gastrointestinal condition, with its prevalence increasing with age [1]. Acute diverticulitis, an inflammatory complication of diverticular disease, affects a significant portion of the population, leading to a substantial healthcare burden [2]. Following an episode of acute diverticulitis, the role of routine colonoscopy has been a subject of considerable debate and evolving clinical guidelines [3]. Historically, colonoscopy was often recommended to exclude underlying colorectal cancer that might be masked by or misdiagnosed as diverticulitis or to identify other significant colonic pathologies [4].

However, recent evidence and updated guidelines increasingly advocate for a more selective, risk-stratified approach to post-diverticulitis colonoscopy [5, 6]. This shift is driven by studies suggesting a low diagnostic yield for colorectal cancer, particularly in patients with uncomplicated diverticulitis, and the desire to avoid unnecessary invasive procedures and their associated risks and costs [7, 8]. The distinction between uncomplicated and complicated diverticulitis, based on radiological and clinical criteria, is crucial in this context, as the risk of underlying malignancy is understood to differ significantly between these two presentations [9].

This study aims to contribute to this ongoing discussion by evaluating the diagnostic yield of colonoscopy in a cohort of patients who experienced acute diverticulitis. Specifically, we sought to assess the relationship between the complexity of diverticulitis (uncomplicated versus complicated) and the findings on subsequent colonoscopy, with a particular focus on the detection of colorectal malignancy. Our objective is to provide further evidence to inform clinical decision-making regarding the appropriate utilization of colonoscopy after an episode of acute diverticulitis, thereby supporting a more targeted and efficient diagnostic strategy.

## Materials and methods

Study design and participants

This retrospective cohort study was conducted at the University Hospital Lewisham, London, England, and included patients diagnosed with acute diverticulitis between January 2022 and May 2025. Patients were identified from the hospital’s electronic health records system. Inclusion criteria comprised adults aged 18 years or older with a confirmed diagnosis of acute diverticulitis based on CT imaging. The CT scan had to clearly demonstrate findings consistent with acute diverticulitis, such as colonic wall thickening, pericolic fat stranding, and/or diverticula. Exclusion criteria included patients with a prior history of colorectal cancer or inflammatory bowel disease or those with incomplete medical records that precluded accurate data extraction for demographic, clinical, radiological, or colonoscopic findings. Patients who did not undergo colonoscopy after diverticulitis were included in the analysis of colonoscopy utilization but excluded from the analysis of colonoscopic findings.

Variables and data sources

Data collected for each patient included demographic information (age, sex), clinical presentation, CT findings, classification of diverticulitis (uncomplicated or complicated), and details of subsequent colonoscopy. Diverticulitis was classified as uncomplicated if characterized by Hinchey stage 1A, localized inflammation without abscess, perforation, fistula, or stricture, and complicated if any of these features were present, based on radiological reports and clinical notes [9]. Colonoscopy findings were categorized as normal mucosa, diverticulosis, benign polyps, strictures, or colorectal cancer. The primary objective of this study was to evaluate the diagnostic yield of colonoscopy following CT-confirmed acute diverticulitis, specifically for the detection of colorectal cancer. Secondary objectives included assessing the association between diverticulitis complexity (uncomplicated vs. complicated) and colonoscopic findings, including benign polyps, strictures, and normal examinations.

Bias

To minimize selection bias, all consecutive patients meeting the inclusion criteria during the study period were considered. Information bias was addressed by using standardized definitions for diverticulitis classification and colonoscopic findings, based on established radiological and pathological reports. Data extraction was performed by three independent researchers, and discrepancies were resolved by consensus.

Study size

A total of 268 patients with CT-confirmed acute diverticulitis were included in the study. Of these, 168 patients (62.7%) underwent a colonoscopy with a median time of 9.1 weeks following their episode of diverticulitis. The sample size was determined by the availability of patient records meeting the inclusion criteria within the specified study period.

Quantitative variables and statistical methods

Descriptive statistics were used to summarize patient characteristics and colonoscopic findings. Categorical variables were presented as frequencies and percentages. A p-value of <0.05 was considered statistically significant. All statistical analyses were performed using Jamovi version 2.7.15 (The jamovi project, Sydney, NSW, Australia).

## Results

Patient characteristics and colonoscopy utilization

A total of 268 patients with CT-confirmed acute diverticulitis were included in the study. Among these, 179 patients (66.8%) presented with uncomplicated diverticulitis, which was defined as Hinchey stage 1a, while 89 patients (33.2%) had complicated diverticulitis, which was defined as Hinchey stage 1b or more. Overall, 168 patients (62.3%) underwent colonoscopy following their episode of diverticulitis. After incorporating the corrected data, the total number of patients who underwent colonoscopy for analysis of findings was 168. The distribution of colonoscopy utilization based on disease complexity is presented in Table 1.

**Table 1:** Distribution of Colonoscopy Utilization by Disease Complexity (n = 268)

Colonoscopy	Uncomplicated (n=179)	Complicated (n=89)
No	65 (36.3%)	36 (40.4%)
Yes	114 (63.7%)	53 (59.6%)

As shown in Table 1, there was no statistically significant diﬀerence in the rate of colonoscopy performance between patients with uncomplicated (114 patients, 63.7%) and complicated (53 patients, 59.6%) diverticulitis (p = 0.510).

Colonoscopic findings

Among the 168 patients who underwent colonoscopy, a diverse range of findings were observed. The most frequent finding was diverticulosis, identified in 137 patients (81.5%). Normal mucosal findings were present in 16 patients (9.5%), indicating no significant pathology. Benign polyps were detected in 10 patients (6.0%), while strictures were noted in four patients (2.4%). A single case of colorectal cancer was identified, accounting for one patient (0.6%) of all colonoscopies performed. The overall distribution of these colonoscopic findings is summarized in Table 2.

**Table 2:** Distribution of Colonoscopic Findings (n = 168)

Finding on Colonoscopy	N	%
Normal	16	9.5
Diverticulosis	137	81.5
Benign Polyps	10	6
Stricture	4	2.4
Cancer	1	0.6

Relationship between complexity and colonoscopic findings

The relationship between the complexity of diverticulitis and the subsequent colonoscopic findings is detailed in Table 3. Of the 168 patients who underwent colonoscopy, 115 patients (68.5%) had a history of uncomplicated diverticulitis, and 53 patients (31.5%) had experienced complicated diverticulitis.

**Table 3:** Relationship Between Complexity and Colonoscopic Findings (n = 168)

Finding on Colonoscopy	Uncomplicated (n=115)	Complicated (n=53)
Normal	14 (12.2%)	2 (3.8%)
Diverticulosis	94 (81.7%)	43 (81.1%)
Benign Polyps	6 (5.2%)	4 (7.5%)
Stricture	1 (0.9%)	3 (5.7%)
Cancer	0 (0.0%)	1 (1.9%)

Notably, colorectal cancer was exclusively identified in the complicated diverticulitis group, with one patient (1.9%) diagnosed with malignancy, while no cases (0.0%) were found in the uncomplicated group. Normal colonoscopy findings were more prevalent in the uncomplicated group (14 patients, 12.2%) compared to the complicated group (two patients, 3.8%). Strictures were observed more frequently in patients with complicated diverticulitis (three patients, 5.7%) than in those with uncomplicated diverticulitis (one patient, 0.9%). The overall association between disease complexity and the spectrum of colonoscopic findings, including benign polyps, was not statistically significant (p = 0.164). This lack of statistical significance is likely attributable to the small number of events for some categories, particularly colorectal cancer, and the overall study size, limiting the inferential power to detect robust associations. Therefore, these findings should be interpreted as hypothesis-generating rather than definitive.

## Discussion

Our study investigated the diagnostic yield of colonoscopy following acute diverticulitis, specifically examining the impact of disease complexity on colonoscopic findings, with a focus on colorectal neoplasia. The findings align with a growing body of literature advocating for a selective, risk-based approach to post-diverticulitis colonoscopy, rather than universal screening [3, 5]; it is important to interpret these results with caution, given the study’s limitations.

Consistent with previous research, our study demonstrated a very low diagnostic yield for colorectal cancer in patients with uncomplicated diverticulitis. No cases of colorectal cancer were identified in the 115 patients (68.5%) with uncomplicated disease who underwent colonoscopy. This observation supports the current trend in guidelines, such as those from the American Gastroenterological Association (AGA), which suggest that colonoscopy may be deferred in patients with uncomplicated diverticulitis, especially if a recent high-quality colonoscopy has been performed [1, 5]. The rationale for this selective approach is to avoid unnecessary invasive procedures, reduce patient burden, and optimize healthcare resource utilization, given the low probability of significant findings in this subgroup [7, 8].

In contrast, our study found that the single case of colorectal cancer was exclusively identified in the complicated diverticulitis group, yielding a cancer detection rate of 1.9% (1/53). While this rate is lower than some reported in meta-analyses (which can range from 5% to 11% for complicated diverticulitis) [9], it underscores the higher risk associated with complicated disease compared to uncomplicated cases. The presence of complications such as abscess, perforation, or stricture may either mask an underlying malignancy or be a direct consequence of a neoplastic process mimicking diverticulitis [4]. Therefore, our results reinforce the recommendation for colonoscopy in patients with complicated diverticulitis, as highlighted by various clinical guidelines [1, 3].

The prevalence of normal colonoscopy findings was higher in the uncomplicated group (12.2%) compared to the complicated group (3.8%), further supporting the notion that uncomplicated diverticulitis is less likely to harbor significant pathology requiring endoscopic intervention. Diverticulosis was the most common finding across both groups, which is expected given the underlying condition. Benign polyps were similarly distributed, suggesting that their occurrence is likely independent of diverticulitis complexity. However, strictures were more frequently observed in complicated diverticulitis, which is a known sequela of severe inflammation and can sometimes be difficult to differentiate from neoplastic strictures without biopsy [9].

Strengths and limitations

Our study possesses several strengths, including its focus on a well-defined cohort of CT-confirmed acute diverticulitis patients and the careful categorization of disease complexity. The incorporation of corrected statistical data ensures a more accurate representation of the findings. However, several limitations warrant careful consideration. The retrospective design inherently introduces potential selection and information biases. Specifically, the fact that only 62.7% (168 of 268 ) of patients underwent colonoscopy raises the possibility of verification bias, where patients with higher perceived risk or more severe symptoms might have been preferentially selected for colonoscopy. This could potentially inflate the observed diagnostic yield in the scoped population. We also acknowledge the lack of adjustment for potential confounding variables such as age, prior colonoscopy history, or the presence of alarm symptoms, which could influence the risk of colorectal neoplasia. The very low number of colorectal cancer cases (n=1) significantly limits the statistical power of our study and makes the conclusions vulnerable to overinterpretation. While the observed trend suggests a higher risk in complicated cases, the overall association between complexity and colonoscopic findings did not reach statistical significance (p = 0.164). This indicates that our findings, while directionally consistent with current literature, should be interpreted cautiously as supportive or hypothesis-generating evidence rather than definitive confirmation. The single-center nature of the study also limits the generalizability of our findings to broader populations. Future prospective, multi-center studies with larger cohorts and standardized colonoscopy referral protocols are warranted to further validate these findings and refine risk stratification models.

In conclusion, our findings support a differentiated approach to colonoscopy after acute diverticulitis. Patients with uncomplicated diverticulitis, particularly in the absence of other risk factors, may not require routine colonoscopic evaluation due to the very low diagnostic yield for colorectal cancer. Conversely, colonoscopy remains crucial for patients with complicated diverticulitis, where the risk of underlying malignancy is demonstrably higher. This selective strategy promotes patient safety, reduces unnecessary procedures, and optimizes healthcare resource allocation. However, the interpretation of these findings must consider the limitations of a retrospective design, potential selection bias, and the statistical non-significance of the association between complexity and colonoscopic findings, particularly given the low event rate of colorectal cancer.

## Conclusions

This cohort study provides further evidence supporting a selective, risk-stratified approach to colonoscopy following acute diverticulitis. Our findings indicate that colonoscopy after uncomplicated diverticulitis has a very low diagnostic yield for colorectal cancer. In contrast, all identified cases of malignancy occurred exclusively in patients with complicated diverticulitis. These results advocate for prioritizing colonoscopic evaluation in patients with complicated diverticulitis or those presenting with additional risk factors for colorectal neoplasia, thereby optimizing diagnostic strategies and resource utilization while minimizing unnecessary invasive procedures for low-risk individuals.
